# Oxaliplatin Antibody-Related Thrombocytopenia: A Case Report

**DOI:** 10.7759/cureus.68621

**Published:** 2024-09-04

**Authors:** Khin Pyai, David I LeRoy, Joseph Attallah, Hosam Hakim, Zyad Kafri

**Affiliations:** 1 Hematology and Medical Oncology, Ascension St. John Hospital, Grosse Pointe Woods, USA; 2 Internal Medicine, Ascension Macomb-Oakland Hospital, Warren, USA; 3 Hematology/Oncology, Ascension St. John Hospital, Grosse Pointe Woods, USA

**Keywords:** thrombocytopenia, folfox, chemotherapy-induced myelosuppression, colorectal cancer, oxaliplatin antibody-mediated thrombocytopenia

## Abstract

Oxaliplatin is used in combination with fluorouracil and leucovorin as part of the FOLFOX (fluorouracil, leucovorin, and oxaliplatin) regimen for colorectal cancer (CRC). Oxaliplatin has been shown to cause thrombocytopenia in a majority of CRC patients receiving this drug. Although this thrombocytopenia mainly occurs through myelosuppression, in rare cases, it can be immune-mediated. However, unlike other chemotherapy-induced myelosuppression, oxaliplatin-induced thrombocytopenia presents with a sudden drop within hours to days of oxaliplatin administration. The majority of cases who present with oxaliplatin-induced thrombocytopenia typically present after actively being treated with oxaliplatin.

Here, we present the case of a 59-year-old female with biopsy-proven CRC on FOLFOX therapy found to have oxaliplatin antibody-mediated thrombocytopenia. She was originally treated with FOLFOX; however, due to response and clinical symptoms, her regimen was changed to include FOLFIRI (leucovorin calcium, fluorouracil, and irinotecan hydrochloride) and bevacizumab before reinitiating FOLFOX due to disease progression. During this case, she presented with rectal bleeding and was found to have severe thrombocytopenia. She was treated with platelet transfusion, intravenous immunoglobulin, and steroids for concerns of immune thrombocytopenia; however, through the use of flow cytometry, oxaliplatin and leucovorin antibodies were discovered. Ultimately, oxaliplatin was permanently discontinued due to concerns about further events of thrombocytopenia.

## Introduction

Oxaliplatin, a third-generation platinum derivative, is a cornerstone of combination chemotherapy regimens such as FOLFOX (fluorouracil, leucovorin, and oxaliplatin). This regimen significantly improves disease-free survival in patients with metastatic or stage II/III colorectal cancer (CRC) [1]. Thrombocytopenia occurs in over 70% of CRC patients who receive oxaliplatin. Grade 3-4 thrombocytopenia leading to life-threatening bleeding is observed in only 3-4% of patients [2,3]. Myelosuppression is the primary cause of oxaliplatin-induced thrombocytopenia, although other factors such as splenic platelet sequestration due to liver damage from oxaliplatin and, in rare instances, immune-mediated thrombocytopenia, have been reported. Unlike chemotherapy-induced myelosuppression, which causes platelet levels to reach nadir 10-14 days after chemotherapy, antibody-mediated thrombocytopenia presents with a sudden drop in platelet count within hours to two days of oxaliplatin administration [3]. Treatment options vary depending on the underlying mechanism. Here, we focus on a case of oxaliplatin antibody-mediated thrombocytopenia.

## Case presentation

A 59-year-old woman with a medical history of hypertension and tobacco dependence was initially diagnosed with liver biopsy-proven Stage IV metastatic colorectal adenocarcinoma with metastases to the lungs and liver. She underwent FOLFOX treatment, completed 12 cycles, and achieved a partial response. Subsequently, she continued with maintenance bevacizumab, which was paused due to severe fatigue. Throughout this period, she had regular follow-ups, including blood tests for carcinoembryonic antigen (CEA) levels and imaging scans with computed tomography (CT) and magnetic resonance imaging (MRI) that revealed a stable disease process. However, after a six-month interruption in bevacizumab treatment, her CEA level showed a slight increase, and an MRI indicated disease progression with an increase in the size of both pulmonary and liver metastases.

Due to disease progression, she started FOLFIRI (leucovorin calcium, fluorouracil, and irinotecan hydrochloride) treatment and completed four cycles. Restaging scans showed evidence of disease progression so she was switched back to FOLFOX treatment. She completed three cycles of FOLFOX without significant issues, except for a minor drop in her platelet count. The fourth cycle was delayed to ensure her platelet levels were stable; however, the day after she received the fourth cycle of FOLFOX, she presented to the emergency department with rectal bleeding. Her platelet count had dropped to less than 2 K/µL from 132 K/µL and her hemoglobin had decreased from a baseline of 11.8 g/dL to 9.1 g/dL within a week. Consequently, she was admitted to the intensive care unit for close monitoring and supportive interventions, which included the administration of two units of platelet transfusion. A dose of intravenous immunoglobulin (IVIG) and a four-day course of 40 mg intravenous dexamethasone were also given due to concerns related to immune thrombocytopenia (ITP). Tests to rule out disseminated intravascular coagulation, hemolysis, and classic ITP were negative or inconclusive; notably, ADAMTS13 activity was within the normal range. Other tests included a peripheral smear and an antibody test for 5-fluorouracil, oxaliplatin, and leucovorin. Pertinent lab results are shown in Table 1.

**Table 1:** Pertinent laboratory values. The table shows laboratory values to help rule out DIC, hemolysis, and classic ITP with reference values. WBC: white blood cell count; LDH: lactate dehyrogenase; PT: prothrombin time; INR: international normalized ratio; PTT: partial thromboplastin time; DIC: disseminated intravascular coagulation; ITP: immune thrombocytopenia

Pertinent labs	Patient’s value	Reference range
WBC	8.1 K/µL	4.0–11.0 K/µL
Absolute reticulocyte count	0.04 Million/µL	N/A
Reticulocytes	1.07%	0.7–1.80%
LDH	306 IU/L	0–240 IU/L
Haptoglobin	98 mg/dL	30–200 mg/dL
PT	12.7 seconds	12.0–14.5 seconds
INR	0.84	N/A
PTT	25.7 seconds	22.5–35.0 seconds
Fibrinogen	357 mg/dL	150–470 mg/dL
Fibrinogen split products	<5	<5
D-dimer	930 ngFEU/mL	<500 ngFEU/mL
ADAMTS-13 activity	63.90%	>66.8%

**Figure 1:**
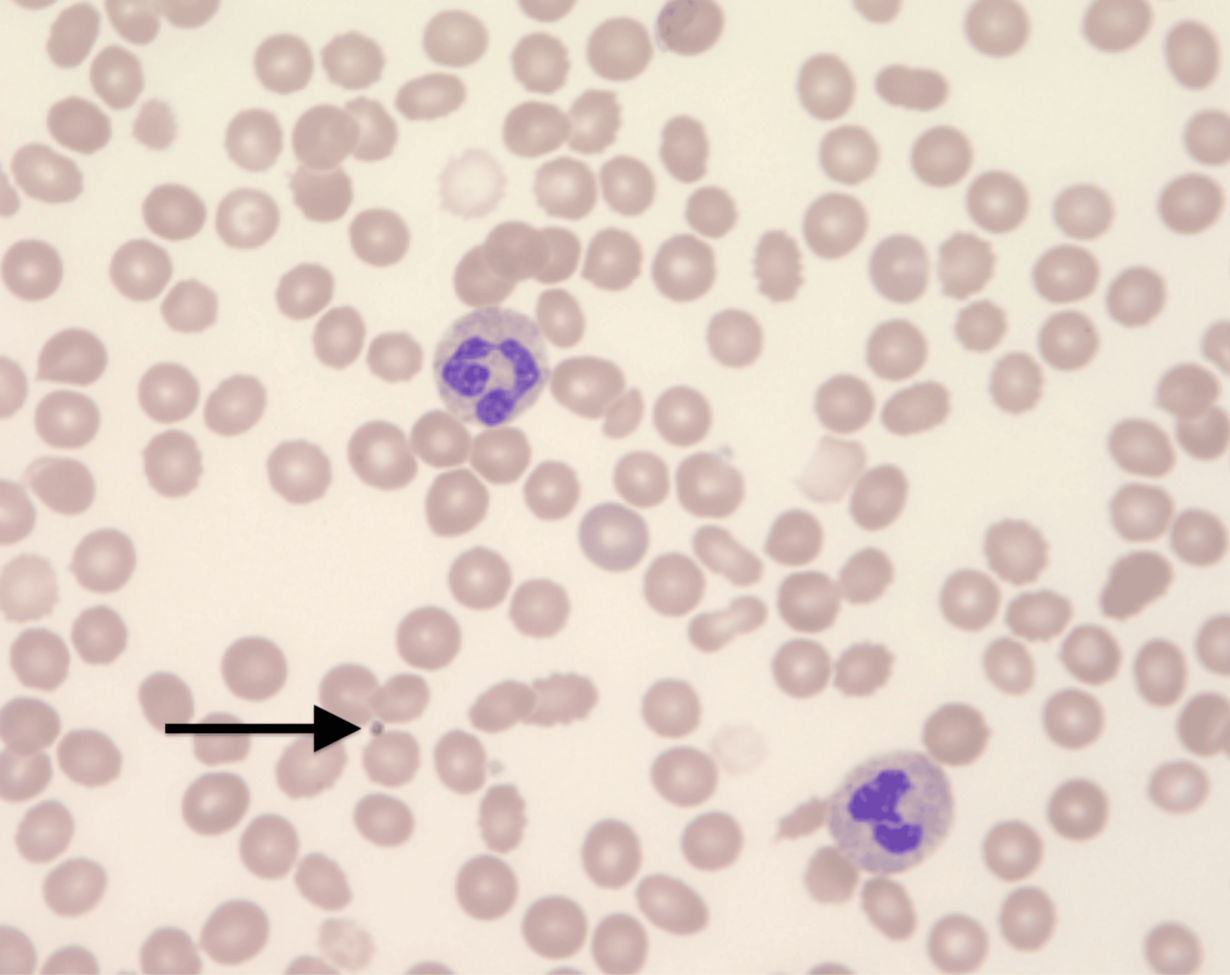
Peripheral blood smear Peripheral blood smear from May 10, 2023. A. Red blood cells: normochromic and normocytic in appearance with mild anisocytosis. B. White blood cells: normal in overall number with a decrease in the absolute number of lymphocytes. C. Platelets: markedly decreased in overall number with no normal morphology; no platelet clumps are noted; arrow pointing to the major finding of one of few platelets seen in the smear, demonstrating the marked decrease in overall number.

The peripheral smear showed mild anisocytosis, normal white blood cell count, a marked decrease in platelet count, normal platelet morphology, and no clumping (Figure 1). Importantly, flow cytometry confirmed the presence of IgG antibodies to both oxaliplatin and leucovorin, strongly suggesting oxaliplatin-induced immune-mediated thrombocytopenia as the cause of her rectal bleeding.

**Figure 2:**
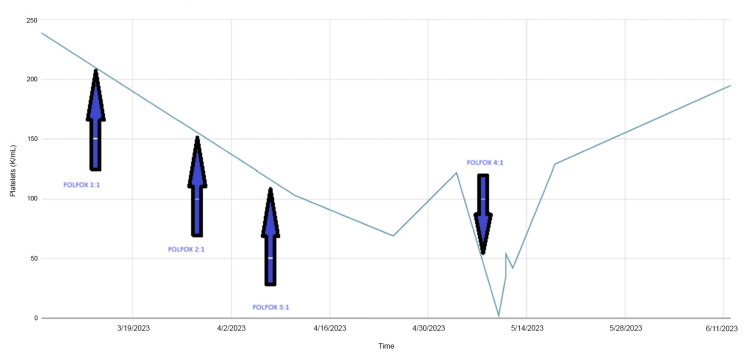
Drop in platelets in correlation with chemotherapy cycles.

After a week of being discharged, her platelet count improved to 129 K/µL (Figure 2). Due to the observed disease progression on the CT scan and the presence of antibody-related thrombocytopenia induced by oxaliplatin, a decision was made to treat her with regorafenib. She continues to follow up with the hematology and oncology clinic, and her platelet counts and hemoglobin levels have remained stable since her discharge.

## Discussion

Thrombocytopenia related to oxaliplatin is a common occurrence, with immune-mediated thrombocytopenia being rare. The basic mechanism responsible for ITP involves antibodies that bind to normal platelets only in the presence of the drug. Oxaliplatin-dependent antibodies against the glycoprotein 2b3a complex are most commonly involved, although the exact immune pathways are not completely understood [4,5]. Currently, hapten-associated antibody response, immune complex formation, or autoantibodies are potential mechanisms for oxaliplatin-dependent antibodies [6]. This condition leads to a sudden and isolated drop in platelet levels within hours of oxaliplatin exposure, with or without clinical bleeding. The platelet count nadir can be as low as 2 K/µL. This form of thrombocytopenia commonly affects females with advanced CRC and a history of prior oxaliplatin exposure, often occurring during retreatment [6]. The majority of cases present after completing at least 10 cycles of oxaliplatin [6]. Hypersensitivity reactions may also occur.

Detecting oxaliplatin-dependent antibodies through flow cytometry is a diagnostic evaluation, but clinical judgment becomes crucial as antibody tests for oxaliplatin may not be readily available at the time of presentation [7-9]. The mainstay of management is definitive discontinuation of oxaliplatin, and platelet counts typically recover within a few days after discontinuation [8]. Supportive measures, including platelet transfusion, are important. While the efficacy of steroids and IVIG has not been proven, they may be considered in cases of sudden-onset thrombocytopenia after oxaliplatin therapy [10]. Additionally, treatment with other chemotherapy agents and biologic agents can be explored as alternatives for the management of the underlying CRC. Other reported cases of oxaliplatin-induced thrombocytopenia with various presentations include hematuria, hematemesis, fevers, chills, and back pains [9]. These reports demonstrated the resolution of thrombocytopenia after discontinuation of oxaliplatin, along with platelet transfusion with and without steroids or IVIG as supportive care [6,9], further illustrating the necessity of discontinuation of oxaliplatin. One study has shown various independent predicting risk factors for chemotherapy-induced thrombocytopenia after oxaliplatin-containing chemotherapy. These factors include total dose of oxaliplatin, baseline platelet count, M stage, albumin, and natural killer cells, which have demonstrated a good predictive effect on risk for thrombocytopenia in patients with CRC treated with oxaliplatin [11].

In our case, the patient completed 12 cycles of FOLFOX during the initial phase of treatment. Later, she received FOLFIRI, but due to disease progression, we had to switch back to FOLFOX. Interestingly, during the retreatment with FOLFOX, we observed a sudden and significant drop in platelet levels within 24 hours of oxaliplatin exposure. Fortunately, her platelet count recovered after providing a supportive platelet transfusion. Due to concerns for future recurrence of thrombocytopenia, oxaliplatin was not rechallenged. We administered IVIG and a four-day course of steroids in response to the challenges in definitively ruling out ITP during the initial phase. However, the exact role and effectiveness of these medications in treating oxaliplatin-induced thrombocytopenia remain uncertain, underscoring the importance of further exploration and research in this field.

## Conclusions

Clinicians need to be aware of the possibility of such an abrupt drop in platelet levels after oxaliplatin administration, as it can be life-threatening. Early detection and timely intervention are crucial to managing this condition effectively. By promptly catching it and implementing supportive measures while discontinuing the drug, we can effectively address the potentially life-threatening consequences. Current research into drug-induced thrombocytopenia is evaluating risk factors for oxaliplatin antibody-mediated thrombocytopenia. However, more research is needed to better understand the mechanisms behind this phenomenon and to identify the most appropriate treatment strategies for patients experiencing oxaliplatin-induced immune thrombocytopenia.

## References

[1] André T, Boni C, Mounedji-Boudiaf L (2004). Oxaliplatin, fluorouracil, and leucovorin as adjuvant treatment for colon cancer. N Engl J Med.

[2] Jardim DL, Rodrigues CA, Novis YA, Rocha VG, Hoff PM (2012). Oxaliplatin-related thrombocytopenia. Ann Oncol.

[3] Woo HS, Lee KH, Yoon PH (2015). Oxaliplatin-induced immune-mediated thrombocytopenia: a case report. Cancer Res Treat.

[4] Bautista MA, Stevens WT, Chen CS, Curtis BR, Aster RH, Hsueh CT (2010). Hypersensitivity reaction and acute immune-mediated thrombocytopenia from oxaliplatin: two case reports and a review of the literature. J Hematol Oncol.

[5] Curtis BR, Kaliszewski J, Marques MB (2006). Immune-mediated thrombocytopenia resulting from sensitivity to oxaliplatin. Am J Hematol.

[6] Erdem GU, Dogan M, Demirci NS, Zengin N (2016). Oxaliplatin-induced acute thrombocytopenia. J Cancer Res Ther.

[7] George JN, Raskob GE, Shah SR, Rizvi MA, Hamilton SA, Osborne S, Vondracek T (1998). Drug-induced thrombocytopenia: a systematic review of published case reports. Ann Intern Med.

[8] Arnold DM, Nazi I, Warkentin TE, Smith JW, Toltl LJ, George JN, Kelton JG (2013). Approach to the diagnosis and management of drug-induced immune thrombocytopenia. Transfus Med Rev.

[9] Stack A, Khanal R, Denlinger CS (2021). Oxaliplatin-induced immune thrombocytopenia: a case report and literature review. Clin Colorectal Cancer.

[10] Aster RH, Bougie DW (2007). Drug-induced immune thrombocytopenia. N Engl J Med.

[11] Li J, Wang W, Jiang K (2024). Risk factors of chemotherapy-induced thrombocytopenia after oxaliplatin-containing chemotherapy for gastrointestinal malignancies. J Gastrointest Cancer.

